# Circulating tumour necrosis factor-α bioactivity in rheumatoid arthritis patients treated with infliximab: link to clinical response

**DOI:** 10.1186/ar1465

**Published:** 2004-12-01

**Authors:** Hubert Marotte, Wlodzimierz Maslinski, Pierre Miossec

**Affiliations:** 1 Departments of Immunology and Rheumatology and Unité Mixte Hospices Civils de Lyon-BioMérieux, Hôpital Edouard Herriot, Lyon, France; 2 Institute of Rheumatology, University of Warsaw, Warsaw, Poland

**Keywords:** TNF, Infliximab, Bioactivity, Response, Treatment

## Abstract

Our objective was to clarify the heterogeneity in response to infliximab treatment in rheumatoid arthritis (RA); to this end, a bioassay was designed to explore the contribution of circulating tumour necrosis factor (TNF)-α bioactivity and its possible link to response. The bioassay is based on the induction of IL-6 and osteoprotegerin (OPG) production by synoviocytes in response to TNF-α. RA synoviocytes were cultured with TNF-α (5 ng/ml) and 42 RA plasma samples collected just before starting therapy. Levels of IL-6 and OPG were measured in supernatants. In 20 of the patients, plasma samples collected before and 4 hours after the first and the ninth infusions were tested in the same way. Plasma concentrations of TNF-α and p55 and p75 soluble receptors were measured using ELISA. TNF-α induced IL-6 and OPG production by synoviocytes, which was further increased with patient plasma dilutions and inhibited by infliximab. With plasma samples obtained before the first infusion, the IL-6-induced production was greater in patients with a good clinical response than in the poor responders (44.4 ± 23.3 ng/ml versus 27.4 ± 20.9 ng/ml; *P *= 0.05). This high circulating TNF-α bioactivity was strongly inhibited with the first infliximab infusion. The difference between IL-6 levels induced with plasma samples obtained before and 4 hours after the first infusion was greater in patients with a good clinical response (40.0 ± 23.7 ng/ml versus 3.4 ± 10.0 ng/ml; *P *= 0.001). Similar findings were obtained for OPG production (7.0 ± 6.2 ng/ml versus 0.0 ± 3.0 ng/ml; *P *< 0.05). Levels of circulating TNF-α bioactivity were predictive of clinical response to TNF-α inhibition, confirming a key role for TNF-α in these RA patients.

## Introduction

Rheumatoid arthritis (RA) is a chronic disease characterized by synovial inflammation that leads to progressive joint damage. Knowledge concerning the role played by cytokines in mediating cell–cell interactions in rheumatoid synovium has led to the rational development of treatment with anticytokine agents. Among these proinflammatory cytokines, tumour necrosis factor (TNF)-α has emerged as a major therapeutic target, based on clinical studies with biological inhibitors such as monoclonal antibodies and soluble receptors. In large proportions of patients, TNF-α inhibitors strongly reduced symptoms of synovitis, biological markers of inflammation and bone destruction [1-4]. However, the improvement varied between patients.

In an attempt to explain these differences between patients, we explored whether heterogeneity exists in the contribution of circulating TNF-α bioactivity, with the hypothesis that patients with higher levels of bioactive TNF-α would be more sensitive to the systemic administration of a specific inhibitor. Such circulating TNF-α activity would reflect local joint production. The goal of the present study was to evaluate circulating TNF-α bioactivity in RA patients before infliximab treatment and to assess its acute modulation by infliximab. Indeed, the remaining TNF-α activity would represent the difference between total TNF-α and its fraction bound to specific and nonspecific inhibitors. Therefore, a bioassay was developed using the properties of synoviocytes to produce IL-6 and osteoprotegerin (OPG) in response to TNF-α [5,6]. Finally, we looked for a possible link between changes in OPG and IL-6 levels and the rate of clinical improvement during infliximab treatment.

## Methods

### Patients

Forty-two patients with RA (35 women and 7 men, median age 46.8 years [range 20–67 years], disease duration 9.0 years [range 1–31 years]), diagnosed according to the revised criteria of the American College of Rheumatology (ACR) [7], were enrolled. Rheumatoid factor was present in 31 of the patients. All received infliximab according to the ATTRACT (Anti-TNF Trial in RA with Concomitant Therapy) protocol at 3 mg/kg every 8 weeks, combined with methotrexate [8]. The following indices were measured: tender joint count, swollen joint count, patient's assessment of pain, patient's global assessment of disease activity, physician's global assessment of disease activity, the Disability Index of the Health Assessment Questionnaire, serum levels of C-reactive protein and erythrocyte sedimentation rate. ACR response was recorded at 54 weeks [9]. RA patients were divided into two groups: good responders, with an ACR response equal to or greater than 50 (*n *= 24); and poor responders, with an ACR response equal to or less than 20 (*n *= 18). EDTA-treated venous blood was collected before infliximab therapy in all patients (*n *= 42). In 20 patients, blood samples were collected during infliximab treatment before and 4 hours after the first and ninth infusions. Plasma samples obtained by centrifugation were stored at -20°C and thawed before use. The main characteristics of the patients are summarized in Table 1.

### Bioassay for circulating TNF-α bioactivity

A functional assay for TNF-α activity was designed using the ability of RA synoviocytes to produce IL-6 and OPG in response to TNF-α [5]. To isolate synoviocytes, RA synovial tissues were finely minced and digested with 4 mg/ml collagenase (Worthington, Freehold, NJ, USA) in phosphate-buffered saline (Life Technologies, Grand Island, NY, USA). These synovium pieces were obtained from RA patients undergoing joint replacement.

Synoviocytes were used at passages four to eight. RA synoviocytes (10^4 ^cells/well) were cultured in 96-well plates (Falcon, Lincoln Park, NJ, USA) in a final volume of 200 μl in minimum essential medium (Life Technologies) supplemented with 10% heat-inactivated foetal calf serum (Life Technologies), 25 000 UI penicillin, 25 000 μg streptomycin and 250 μg fungizone.

TNF-α (0–10 ng/ml) was added to RA synoviocytes with or without infliximab (10 μg/ml). Then, TNF-α (5 ng/ml) was combined with 20 μl plasma per well in order to increase the sensitivity of the synoviocyte response. Plasma samples were collected just before the first infusion. In addition, RA synoviocytes were stimulated with plasma samples collected before and 4 hours after the first and the ninth infusions from 20 RA patients. TNF-α (5 ng/ml), with or without infliximab (10 μg/ml), was preincubated with these four plasma samples for 1 hour before being added to the culture.

IL-6 and OPG production were measured by ELISA in 48-hour supernatants, as previously described [5,6]. At baseline, in the 20 patients with a 1-year follow up, plasma concentrations of TNF-α p55 and p75 soluble receptors were measured using commercial ELISA kits (Biosource, Camarillo, CA, USA), in accordance with the manufacturer's instructions.

### Statistical analysis

Statistical analysis was performed using the Statview software (Abacus Concept Inc., Cary, NC, USA). Means were compared using a nonparametric test. Spearman's correlation was used to determine a relationship between the changes in IL-6 and OPG levels and those of TNF-α or TNF soluble receptors detected by ELISA. χ^2 ^test was performed to detect differences between different subsets.

## Results

### Principle of the bioassay

**Figure 1:**
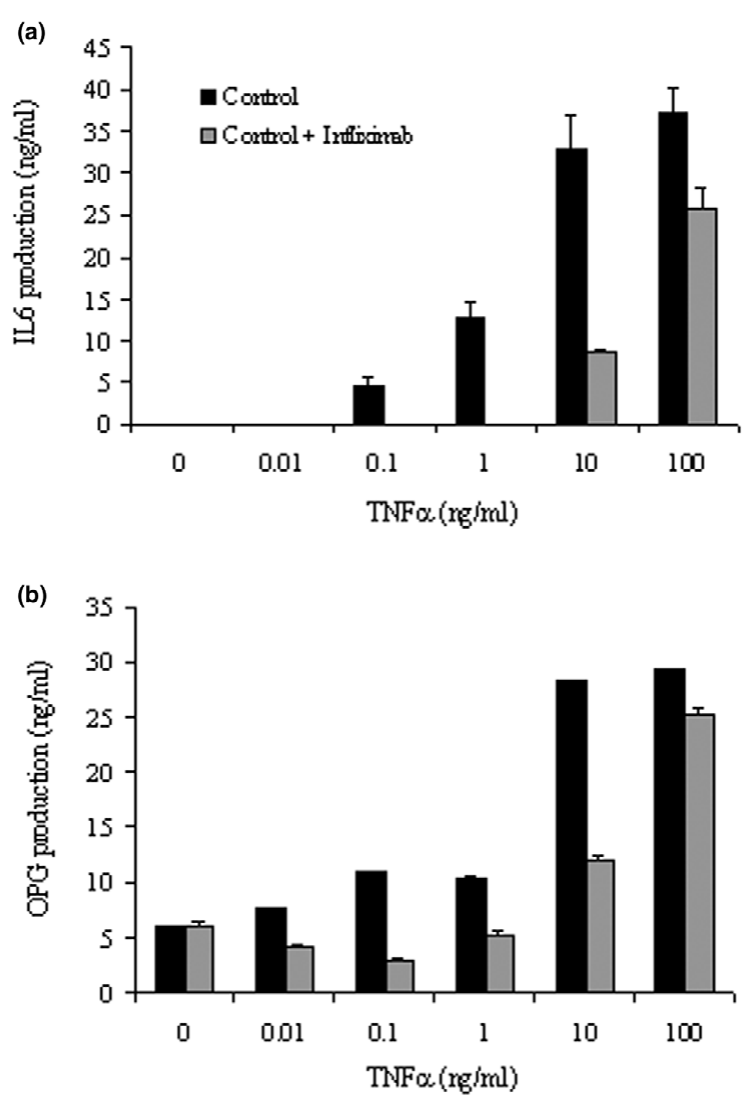
Principle of the bioassay. Rheumatoid arthritis synoviocytes (10^4 ^cells/well) were cultured in 96-well plates and stimulated with increasing doses of tumour necrosis factor (TNF)-α (0–100 ng/ml). Levels of **(a) **IL-6 and **(b) **osteoprotegerin (OPG) were measured in 48-hour supernatants. Infliximab at 10 μg/ml was preincubated for 1 hour with TNF-α before its addition to the culture.

The principle of the bioassay, as shown in Fig. 1, is based on the ability of TNF-α to induce IL-6 and OPG production by RA synoviocytes. With TNF-α concentrations ranging from 0.1 to 100 ng/ml, IL-6 production by synoviocytes increased in a dose-dependent manner. Addition of infliximab at 10 μg/ml completely inhibited the effect of TNF-α at 1 ng/ml, and reduced that of TNF-α at 10 ng/ml by 74% (32.9 ng/ml without versus 8.5 ng/ml with infliximab; Fig. 1a). Similar studies were performed for OPG production. As for IL-6, OPG production by synoviocytes increased in a dose-dependent manner in response to TNF-α (Fig. 1b). Maximal concentrations of IL-6 and OPG were in the same range up to 35 ng/ml. With regard to IL-6, addition of infliximab inhibited OPG production induced by TNF-α at 10 ng/ml.

**Figure 2:**
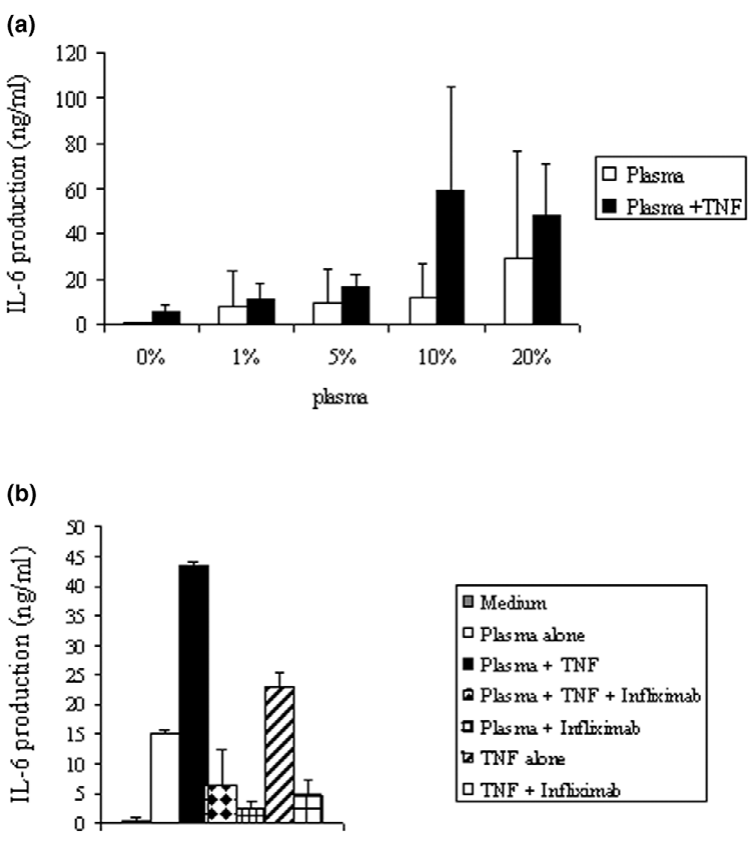
Pattern of plasma tumour necrosis factor (TNF) bioactivity in rheumatoid arthritis (RA) patients and healthy individuals. Using the ability of TNF-α to stimulate RA synoviocytes, TNF-α (5 ng/ml) was combined with increasing concentrations of plasma from RA patients (0–20%). **(a) **Four RA plasma samples were obtained before infliximab therapy. **(b) **RA plasma samples (*n *= 2) were used at 10% dilution. IL-6 production was measured by ELISA in 48-hour supernatants.

Addition of plasma further increased the effect of exogenous TNF-α. With RA plasma concentrations ranging from 0% to 20% used alone (*n *= 4), IL-6 production by synoviocytes increased in a dose-dependent manner (Fig. 2a). This effect was further increased when exogenous TNF-α at 5 ng/ml was added. The greatest effect was observed with 10% plasma. An inhibitory effect was often observed with concentrations of plasma at 20%. In further experiments, 5 ng/ml TNF-α and 10% plasma concentration were used. With RA plasma samples collected before infliximab therapy used alone, IL-6 production was 6.5 ± 4.8 ng/ml (*n *= 20).

The contribution of TNF bioactivity is shown in Fig. 2b. Combination of 10% concentration of RA plasma with TNF-α at 5 ng/ml increased IL-6 production to 43.5 ng/ml. This effect was inhibited by infliximab (6.5 ng/ml), demonstrating the specificity for TNF-α.

### Effect of patient plasma samples collected just before the first infliximab infusion

**Figure 3:**
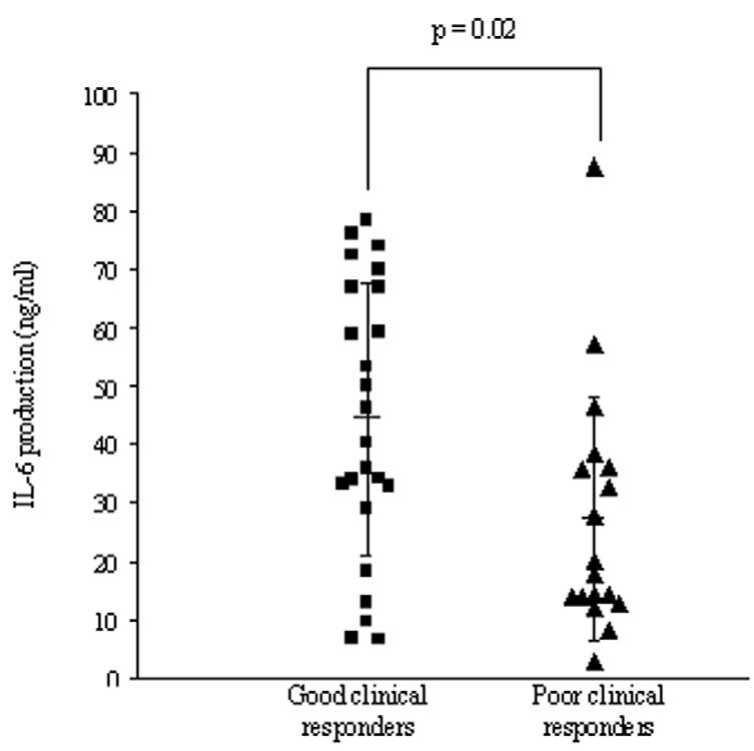
Link between circulating tumour necrosis factor (TNF)-α bioactivity and clinical response. Using the ability of TNF-α to stimulate rheumatoid arthritis synoviocytes, TNF-α (5 ng/ml) was combined with 20 μl plasma per well in order to increase the sensitivity of synoviocyte response. Levels of circulating TNF-α bioactivity were estimated with plasma samples obtained before infliximab therapy and separated according to American College of Rheumatology (ACR) clinical response (good or poor) at 54 weeks (*n *= 42). A good clinical response was defined as an ACR 50 response or better (*n *= 24).

Samples were obtained from 42 patients before the first infliximab infusion. The levels of IL-6 produced by stimulation with 10% plasma and TNF-α (5 ng/ml) are shown in Fig. 3, stratified by ACR response observed at week 54. Levels were higher for the good responders (ACR response ≥ 50) than for poor responders (ACR ≤ 20; 44.4 ± 23.3 ng/ml versus 27.4 ± 20.9 ng/ml; *P *= 0.05).

### Circulating TNF-α bioactivity modulation by the first infliximab infusion

For 20 of the patients, samples were collected before and after the first and the ninth infusions. There was no significant clinical difference between the 20 patients and the rest of the 42 patient cohort (Table 1).

**Figure 4:**
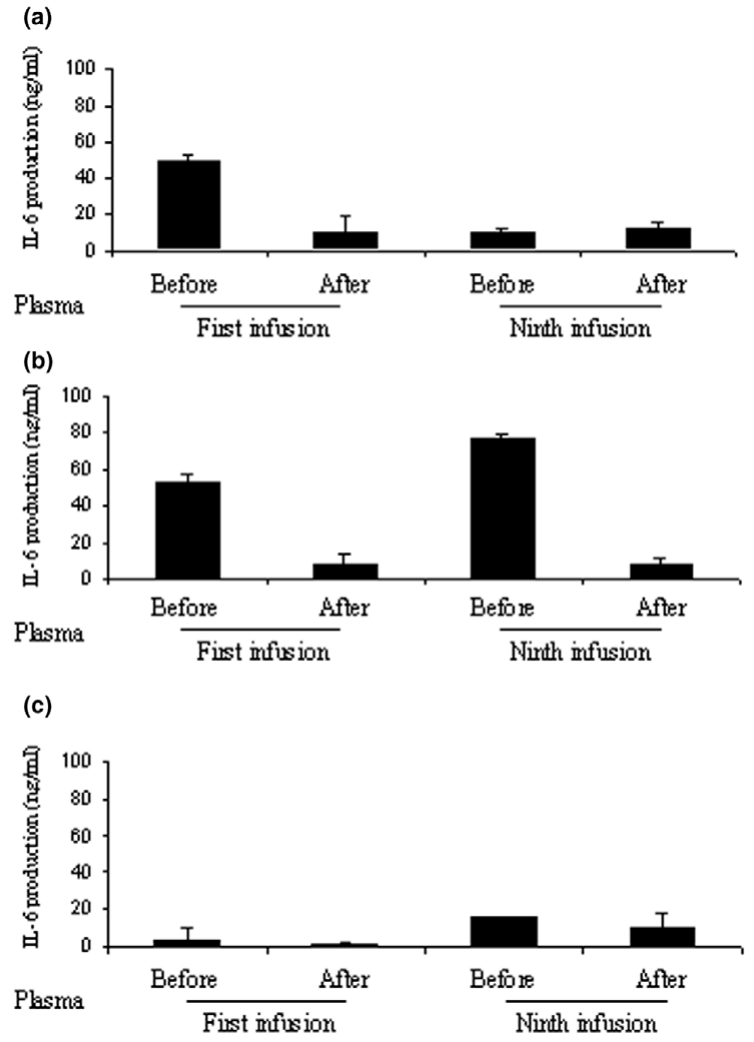
Patterns of plasma tumour necrosis factor (TNF)-α bioactivity in rheumatoid arthritis (RA) patients treated with infliximab. Using this bioassay three patterns were observed and linked to the clinical response in the 20 patients with a 1-year follow up. **(a) **The first pattern showed good ability of the plasma to induce IL-6 production before infliximab therapy, followed by complete inhibition 4 hours after the first infusion. Plasma samples before the ninth infusion had low IL-6-inducing activity. This pattern was associated with an American Colege of Rheumatology (ACR) 50 response at 54 weeks (*n *= 6). **(b) **In the second pattern the effect of the first infusion was similar to that in the first pattern. However, high IL-6 inducing activity was still present before the ninth infusion but remained sensitive to treatment. In this pattern, five patients had an ACR 50 response but one had no response. **(c) **In the last pattern the first plasma samples had a moderate or no effect on IL-6 production. This activity was not sensitive to infliximab infusion. All patients (*n *= 8) had a poor clinical response (ACR <20). A link was observed between these patterns and clinical response (χ^2 ^= 16.6; *P *< 0.001).

The high IL-6-inducing activity found in samples before treatment was strongly reduced in samples obtained 4 hours after the first infusion, following TNF-α /anti-TNF-α complex formation induced by the infliximab infusion (Fig. 4a,4b). This reduction demonstrates the contribution of TNF-α to the activity following *in vivo *administration of the TNF-α inhibitor. This pattern of reduction was associated with a very good clinical response at 54 weeks for all patients (ACR 50; *n *= 11) except one (ACR 20). Conversely, no modulation in circulating bioactivity was observed in patients with a low level of TNF-α bioactivity (Fig. 4c). This pattern was associated with a poor clinical response (ACR <20) except in one patient (ACR 50).

**Figure 5:**
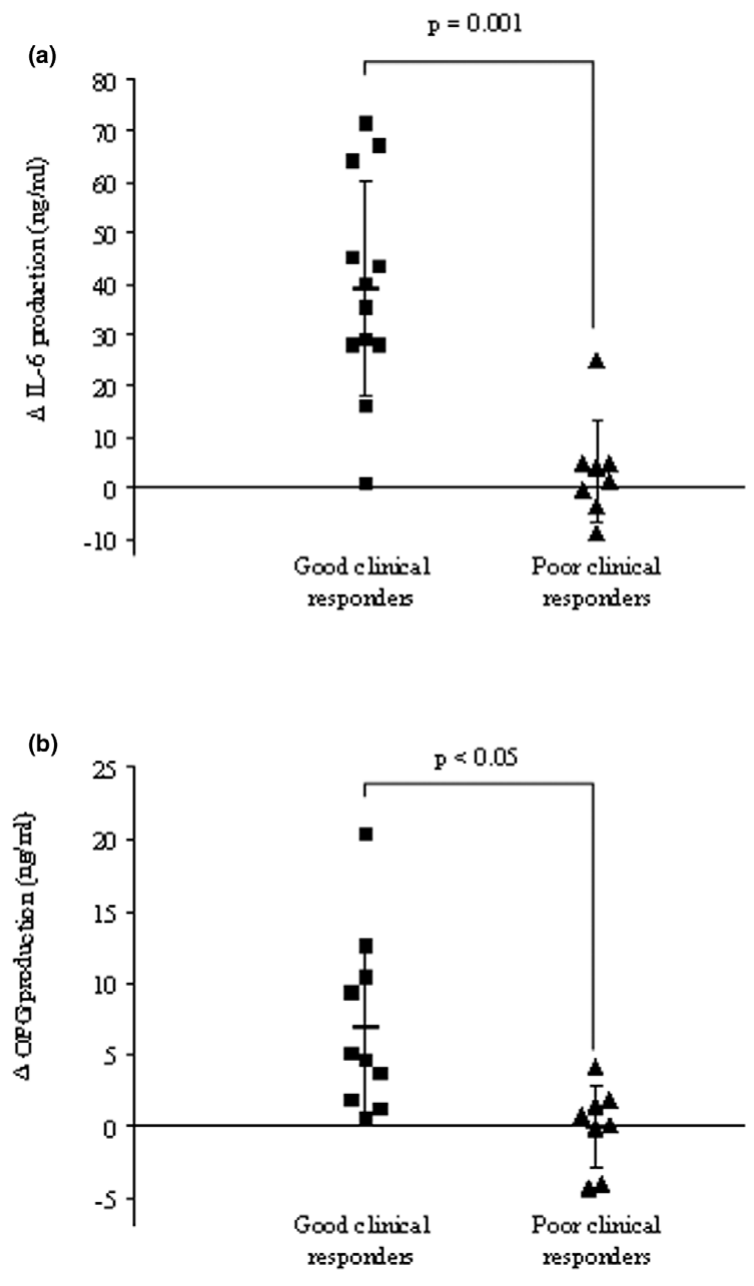
Link between changes in IL-6 and osteoprotegerin (OPG) production by rheumatoid arthritis (RA) synoviocytes and clinical response. Changes, expressed as ΔIL-6 and ΔOPG production by RA synoviocytes before and 4 hours after the first infliximab infusion, are separated according to the American College of Rheumatology (ACR) clinical response (good or poor) at 54 weeks (*n *= 20). A good clinical response was defined as an ACR 50 response or better (*n *= 12).

The difference between the IL-6 levels induced with TNF-α at 5 ng/ml and plasma obtained before and 4 hours after the first infusion correlated with good clinical response (40.0 ± 23.7 ng/ml versus 3.4 ± 10.0 ng/ml; *P *= 0.001; Fig. 5a). Similarly, OPG levels were measured in the same supernatants. With regard to IL-6, the difference between the levels of OPG with plasma samples obtained before and 4 hours after the first infliximab infusion correlated with good clinical response (7.0 ± 6.2 ng/ml versus 0.0 ± 3.0 ng/ml; *P *< 0.05; Fig. 5b). A positive correlation was observed between changes in IL-6 and OPG production (*n *= 20; r = 0.843; *P *< 0.001).

### Circulating TNF-α bioactivity modulation by the ninth infusion

Patients with high circulating bioactivity at the first infusion could be separated into two subsets. For the first group, before the ninth infusion, no circulating bioactivity was detected (Fig. 4a). This pattern was associated with very good clinical response at 54 weeks in all patients (ACR 50; *n *= 6). In the second group, circulating bioactivity was still present before the ninth infusion but remained sensitive to further infliximab administration (Fig. 4b). With this pattern, five out of six patients had an ACR 50 response but one had a poor response (ACR ≤ 20). No modulation in circulating bioactivity was observed in patients with low circulating TNF-α bioactivity before infliximab therapy (*n *= 8; Fig. 4c). A link was observed between these patterns and the clinical response (χ^2 ^= 16.6; *P *< 0.001).

Various clinical and biological parameters were analyzed according to these patterns. Before treatment, no difference was observed between joint counts, erythrocyte sedimentation rate, C-reactive protein, rheumatoid factor positivity, or Disease Activity Score 28 (5.4 ± 0.9 in good responders versus 5.2 ± 1.4 in poor responders; *P *= 0.6).

### Absence of correlation between circulating TNF-α bioactivity and ELISA levels of TNF-α or soluble receptors

Levels of TNF-α and of soluble receptors (p55 and p75) were measured in plasma samples obtained at baseline from 20 RA patients. The mean TNF-α level was 144.87 ± 130.36 pg/ml in good responders and 153.75 ± 132.93 pg/ml in poor responders (*P *= 0.97). Similar results were observed with p55 (2534 ± 1074 pg/ml versus 2436 ± 953 pg/ml; *P *= 0.57) and p75 (3054.8 ± 673.8 pg/ml versus 2332.2 ± 921.3 pg/ml; *P *= 0.84) soluble receptors in good responders versus poor responders. No correlation was observed between circulating TNF-α bioactivity and TNF-α or soluble receptors, or the difference between TNF-α and p55 and p75 levels. Similarly, no negative correlation was observed between levels of circulating TNF-α bioactivity and of p55 or p75 soluble receptors.

## Discussion

Prediction of the response of RA to treatment remains a hot topic. There is no evidence that simple determination of plasma TNF-α levels by ELISA allows such prediction for treatment with TNF-α inhibitors. However, it still makes sense that patients producing high levels of TNF-α will be more sensitive to TNF-α inhibition.

A system was established to evaluate the circulating TNF-α-related bioactivity in plasma. Exogenous TNF-α alone stimulates IL-6 production and this effect can be abrogated by first incubating TNF-α with infliximab before exposure to the synovial cells. When plasma is combined, cells respond to free TNF-α and not to inactive TNF-α bound to specific soluble receptors and to other less specific binding sites on proteins. This bioassay was based on the IL-6 production induced by the combination of TNF-α and plasma. Addition of exogenous TNF-α was used to detect the presence of circulating inhibitors. Such inhibitors in plasma are probably involved in the lower effect of 20% plasma concentration as compared with the effect seen with a 10% concentration (Fig. 2a).

When the system was applied to explain part of the heterogeneity in treatment response, a third of the patients showed strong and prolonged inhibition in circulating TNF-α bioactivity, suggesting the critical contribution of systemic TNF-α in these patients. In another third of the patients circulating TNF-α bioactivity was inhibited by infliximab infusion for a short time, because bioactive TNF-α reappeared but was again inhibited by the next infliximab infusion. This profile suggested partial inhibition, although the clinical benefit was still very significant. In two thirds of the samples, the bioassay measured a strong inhibition of circulating TNF-α-related activity during the first infusion. The link between strong anti-TNF-α activity induced by the first infusion and the good clinical response confirms the key role played by TNF-α in approximately two thirds of the RA patients. This result is in accord with results from the ATTRACT study [8]. In contrast, in the last third of the patients the assay suggested no contribution or a reduced contribution of TNF-α bioactivity either before or after the first infusion of infliximab. All plasma samples from these patients inhibited the IL-6 production usually induced by TNF-α. This profile suggests that these patients may have a high level of innate neutralizing TNF-α activity and/or no circulating active TNF-α. Patients with this pattern exhibited a poor clinical response, suggesting that their RA was not much driven by TNF-α activity alone but probably by other cytokines or mechanisms.

The heterogeneity in these patterns may be explained by higher disease activity for responders than nonresponders, but no difference was observed in clinical and biological parameters before treatment. One way to view the difference among patients with high circulating TNF-α bioactivity initially is the link observed between higher trough concentrations of infliximab in RA patient serum and good response and a reduced progression of radiographic joint damage [10]. This latter study suggested that RA patients with a poor clinical response tend to eliminate infliximab more rapidly from their circulation.

RA synoviocytes produce higher levels of OPG than do peripheral blood mononuclear cells or synovial fluid mononuclear cells, but they do not produce soluble receptor activator of nuclear factor-κB ligand [5]. Accordingly, we focused on OPG, which is produced in response to TNF-α. Similar results were observed for IL-6 and OPG production, although changes in IL-6 levels appeared more sensitive. This is related to the very low levels of IL-6 produced by resting synoviocytes. Accordingly, the predictive value of changes in IL-6 was better than that of OPG. Our findings extended previous studies indicating a correction in high OPG serum levels in RA patients treated with infliximab [5].

No correlation was observed between circulating TNF-α bioactivity and its protein concentration in plasma measured by ELISA. Circulating TNF-α bioactivity levels could not be calculated as free protein TNF-α taking into account the levels of TNF-α and soluble receptors (p55 and p75) measured by ELISA. This discrepancy further indicates the usefulness of a bioassay when function is the key. Such complexity has been observed when trying to explain loss of infliximab response by the induction of anti-mouse antibodies [11]. Once again, only the demonstration of inhibitory activity *in vivo *would allow such a conclusion to be drawn.

## Conclusion

In conclusion, this bioassay was able to predict correctly the clinical response in 69% of cases (29/42). Taking into account the effect of the first infusion increased the value to 90%. However, a simplified assay would need to be designed for routine application.

## Abbreviations

ACR = American College of Rheumatology; ELISA = enzyme-linked immunosorbent assay; IL = interleukin; OPG = osteoprotegerin; RA = rheumatoid arthritis; TNF = tumour necrosis factor.

## Competing interests

The author(s) declare that they have no competing interests.

## Authors' contributions

HM conducted the experiments and wrote the paper. WM took OPG measurements. PM directed the research.

## Figures and Tables

**Table 1:** Patient characteristics

Characteristic	All RA patients (*n *= 42)	RA patients with a 1-year follow up (*n *= 20)	*P*
Age (years)	46.81 ± 10.78	48.05 ± 9.47	0.51
Sex (% female)	83 ± 11.27	70 ± 20.08	0.38
Disease duration (years)	8.98 ± 8.39	8.80 ± 8.01	0.82
Previous DMARD treatment (*n*)	2.78 ± 1.41	2.70 ± 2.70	0.47
Swollen joint count (0–28)	4.57 ± 3.16	4.55 ± 2.70	0.43
Tender joint count (0–28)	9.21 ± 5.78	8.35 ± 6.07	0.80
DAS28 score	5.33 ± 1.12	5.31 ± 1.19	0.76
ESR (mm/hour)	37.59 ± 23.18	37.45 ± 23.71	0.91
CRP (mg/l)	26.93 ± 24.87	31.30 ± 27.24	0.65

Values are expressed as mean ± standard deviation. CRP, C-reactive protein; DAS28, Disease Activity Score 28; DMARD, disease-modifying antirheumatic drug; ESR, erythrocyte sedimentation rate; RA, rheumatoid arthritis.
